# CaZF, a Plant Transcription Factor Functions through and Parallel to HOG and Calcineurin Pathways in *Saccharomyces cerevisiae* to Provide Osmotolerance

**DOI:** 10.1371/journal.pone.0005154

**Published:** 2009-04-13

**Authors:** Deepti Jain, Nilanjan Roy, Debasis Chattopadhyay

**Affiliations:** 1 National Institute of Plant Genome Research, Aruna Asaf Ali Marg, New Delhi, India; 2 National Institute for Pharmaceutical Education and Research, SAS Nagar, Punjab, India; University of Massachusetts Amherst, United States of America

## Abstract

Salt-sensitive yeast mutants were deployed to characterize a gene encoding a C2H2 zinc finger protein (CaZF) that is differentially expressed in a drought-tolerant variety of chickpea (*Cicer arietinum*) and provides salinity-tolerance in transgenic tobacco. In *Saccharomyces cerevisiae* most of the cellular responses to hyper-osmotic stress is regulated by two interconnected pathways involving high osmolarity glycerol mitogen-activated protein kinase (Hog1p) and Calcineurin (CAN), a Ca^2+^/calmodulin-regulated protein phosphatase 2B. In this study, we report that heterologous expression of CaZF provides osmotolerance in *S. cerevisiae* through Hog1p and Calcineurin dependent as well as independent pathways. CaZF partially suppresses salt-hypersensitive phenotypes of *hog1*, *can* and *hog1can* mutants and in conjunction, stimulates HOG and CAN pathway genes with subsequent accumulation of glycerol in absence of Hog1p and CAN. CaZF directly binds to stress response element (STRE) to activate STRE-containing promoter in yeast. Transactivation and salt tolerance assays of CaZF deletion mutants showed that other than the transactivation domain a C-terminal domain composed of acidic and basic amino acids is also required for its function. Altogether, results from this study suggests that CaZF is a potential plant salt-tolerance determinant and also provide evidence that in budding yeast expression of HOG and CAN pathway genes can be stimulated in absence of their regulatory enzymes to provide osmotolerance.

## Introduction

Plants have to cope with several types of environmental stress conditions. Water deficit, salty soils and cold are the common stress conditions affecting plant development [1]. Among them, high salinity is one of the most serious limiting factors in plant growth and productivity [1]. Cells constantly evaluate and respond to sudden and adverse changes in environment by certain mechanisms that not only initiate the repair of macromolecular damage but also establish a tolerant state, which helps to prevent further damage. Budding yeast (*Saccharomyces cerevisiae*) has been considered an excellent model for the study of the mechanisms underlying tolerance, particularly to saline stress [2], because of the high degree of evolutionary conservation of stress pathways between higher eukaryotes and *S. cerevisiae* and the ease with which yeast genes encoding components of the pathways can be manipulated.

In high osmotic condition, *S. cerevisiae*, initiates an efficient adaptive response, which maintains cellular Na^+^/K^+^ balance, retains turgor and repairs cellular damages. Principally, two interconnected pathways regulate this adaptive response. Elevated cytosolic Ca^2+^ activates Calcineurin (CAN) due to extracellular hyperionic stress, a heterodimeric phosphatase 2B with two catalytic subunits, CNA1 and CNA2, and a regulatory subunit CNB. It then dephosphorylate a C2H2 zinc finger transcription factor CRZ1/TCN1 [3], [4] causing its transport to nucleus to activate expression of a P-type ATPase ENA1/PMR2A for Na^+^ and Li^+^ efflux [5], but only a part of ENA1 expression is CAN-dependent [4] suggesting that other Na^+^-stress response pathways also contribute to ENA1 induction [6]. Calcineurin mutants (i.e., *cna1cna2* and *cnb*) fail to grow in growth medium having high concentration of either Na^+^, Li^+^, or Mn^2+^ [7]–[9] suggest that CAN participates in regulating the intracellular concentration of several ions [10], [11]. In addition to ENA1, some other gene(s) are also contributing to salt tolerances that have been regulated by calcineurin osmopathway [12].

The high osmolarity glycerol (HOG) pathway is regulated by a mitogen activated protein kinase (MAPK) Hog1p [13], [14]. Drastic reduction of osmotolerance in the *hog1* mutants demonstrates the essentiality of this module in hyperosmotic stress. At least two osmosignalling branches, through a series of downstream components, activate MAPK kinase Pbs2p, which in turn phosphorylate and activates MAPK, Hog1p [15]–[17]. Activated Hog1p after moving to nucleus further induces downstream osmoresponsive genes through at least five transcription regulators. Msn2p, Msn4p [18]–[20] are two functionally redundant C2H2 zinc finger proteins and activate STRE (Stress responsive upstream activator element) mediated induction of several general stress responsive genes CTT1, HSP12, DDR2, TPS2 etc, required possibly for damage repair [21]–[23]. Two other Hog1p-regulated transcription activators, Msn1p and Hot1p regulate GPD1, GPP2, genes for glycerol biosynthesis enzymes [19]. Under osmotic stress Hog1p regulated transcription factors recruit activated Hog1p directly to osmoresponsive promoters [24], [25] that further stimulate recruitment of RNA Pol II [26] and Rpd3 histone deacetylase to promote transcription initiation [27]. Sko1p [28], related to bZIP/ATF family of transcriptional regulators [29], represses *ENA1* expression through CRE (Cyclic AMP Responsive Element) in unstressed condition. Under hypertonic stress Sko1p is phosphorylated by Hog1p and converted into a transcription activator by recruiting SAGA histone deacetylase and SWI/SNF complex to promote chromatin remodeling [25] and induce *ENA1* expression in conjunction with Calcineurin/Crz1p mediated pathway [28].

Research over the past decade has identified several cellular mechanisms of salt tolerance in yeast that are conserved in plant cells; and isolation, and characterization of a number of plant salt tolerance determinants was based on homologous function [30]–[35] in yeast. Calcium sensor-regulated stress response pathways seem to be structurally and functionally conserved in plants [36]–[38] and some abiotic stress-related proteins are often found to functionally complement yeast calcineurin knockouts. In tobacco and *Arabidopsis* NACK-PQR pathway, similar to HOG pathway, have been reported [39]. Tobacco MAPK kinase NQK1 can functionally complement Pbs2p [39].

As drought and high salinity are amongst the major challenges for plant survival, our interest is focused on one chickpea (*Cicer arietinum*) gene highly expressed in a drought tolerant cultivar in comparison to a drought sensitive cultivar in response to drought and provided tolerance to high salt when expressed in tobacco. The gene, *CaZF* encodes a C2H2 zinc finger protein. As zinc finger proteins are ubiquitous; and drought and salt stress share some common signaling pathways we decided to investigate if there is any osmoregulatory response mediated by CaZF in *S. cerevisiae*, in an attempt to outline the *in vivo* function of chickpea CaZF. Overexpression of *CaZF* cDNA in a galactose-inducible manner in yeast demonstrated that CaZF is able to rapidly improve salt tolerance of yeast cells under saline stress. Moreover, CaZF is able to complement osmotolerance deficiencies in *hog1*, *cnb1*, and *hog1cnb1* double mutants concomitantly with an increased accumulation of osmolyte glycerol and stress-responsive genes regulated by Hog1p and CAN.

## Results and Discussion

### Differential expression of *CaZF*, a gene for C2H2 zinc finger protein from chickpea

**Figure 1:**
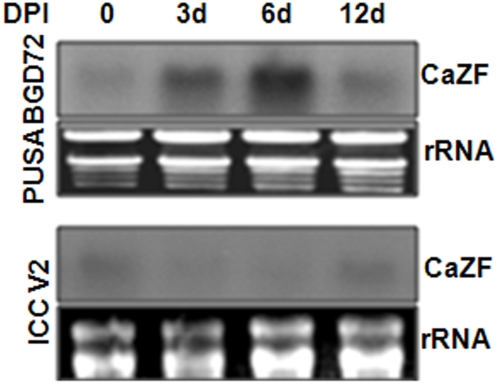
Expression of CaZF in chickpea varieties BGD72 and ICCV2 under different drought conditions. Samples harvested at day post-irrigation (DPI) is mentioned. Total RNA (20 µg/lane) from chickpea seedlings were hybridized with probe prepared from *CaZF* cDNA as described under “Experimental Procedures”. Ribosomal RNAs (rRNA) are shown as loading control.

**Figure 2:**
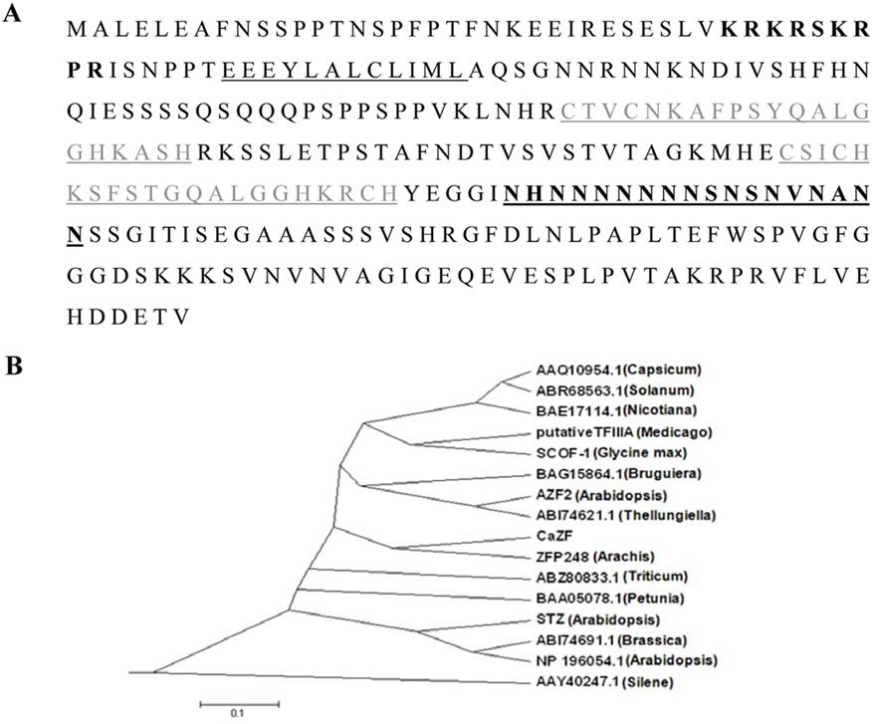
In silico analysis of CaZF protein. *A*, Deduced amino acid sequence of CaZF. The basic B-box in CaZF is indicated by *bold letters*, L-box by an *underline*, zinc finger motifs by *underlined grey letters* and Asn-rich region by *underlined bold letters*. *B*, Phylogenetic tree showing relationship between CaZF and other well-studied C2H2 zinc-finger family proteins. The tree was generated using the neighbor-joining algorithm of MEGA 2.0 software, version 2.1. The bar indicates the scale for branch length.

Subtracted cDNA libraries constructed between two chickpea (*Cicer arietinum*) cultivars at different points of drought-stress resulted in a number of EST clones expressing higher in the drought tolerant BGD72 than in the sensitive ICCV2 in response to drought. An EST encoding a putative zinc finger protein expressing more in BGD72 than in ICCV2 at different points of stress (Figure 1) was taken for further studies. Full-length cDNA (*CaZF*) constructed by 5′ RACE was 1185 bp in length (GenBank accession EU513298). Sequence analysis revealed an 843 bp open reading frame (ORF) of 280 amino acid, 139 bp long 5′ and 203 bp long 3′ untranslated region. Deduced amino acid sequence shows (Figure 2A) CaZF is an EPF type C2H2 zinc finger protein having two canonical TFIIIA-type zinc finger motifs (CX_2_CX_3_FX_5_LX_2_HX_3_H). Both the zinc finger motifs contain conserved QALGGH sequence. A short spacer sequence of 28 amino acids separates two zinc fingers. Among the studied proteins PIF1 (GB: AAQ54302), a pathogen inducible zinc finger protein from capsicum shows maximum sequence similarity with CaZF of only about 55% homology (expect = 5e-45). Notably, PIF1 is also highly expressed in a pathogen tolerant variety compared to a sensitive one in response to infection [40]. Detailed comparisons of the amino acid sequences among plant zinc finger proteins revealed three conserved regions other than the zinc fingers. CaZF contains a short basic region with a consensus of KXKRSKRXR (B-box), near the N-terminus, which may function as a potential nuclear localization signal (NLS) and/or may participate in DNA binding. Another is a region, consisting of three acidic residues followed by hydrophobic residues rich in leucine, with a consensus of EXEXXAXCLXXL (L-box) located between B-box and the first zinc-finger. The other is a short hydrophobic region containing a highly conserved DLNL sequence as a core (DLN-box) close to the C-terminus. The latter two may play a role in protein-protein interactions or in maintaining the folded structure. CaZF possesses a serine-glutamine rich region at the N-terminus, between L-box and first zinc-finger, which might function as a transactivation domain as suggested for ZPT2-1 and Pszf1 [41], [42] or might be a phosphorylation site for post-translational modification; and an asparagine rich stretch after the second zinc finger at the C-terminus. Similar asparagine-rich domains are also present in some stress-inducible zinc finger proteins such as SCOF-1, EPF2-5, and STZ [43]. Like *STO* and *STZ*, the Arabidopsis cDNAs, which increase salt tolerance in yeast in a Calcineurin independent manner, SCOF-1 and EPF2-5, CaZF contains highly basic region followed by acidic amino acids near the C-terminus. But, CaZF has two such combinations of basic and acidic amino acid stretches. Phylogenetic analysis showed that CaZF and one Arachis protein (ZFP248) shares the same clad (Figure 2B).

### CaZF binds in vitro to EP sequence repeat and activates transcription in yeast

**Figure 3:**
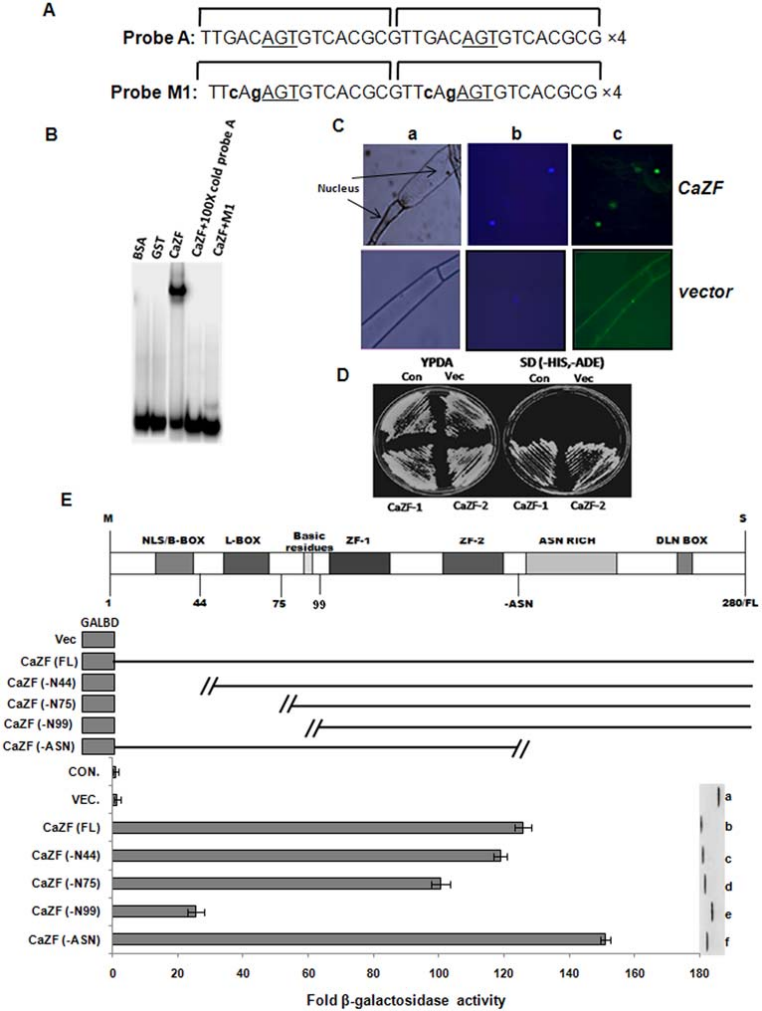
DNA binding, cellular localization and transactivation assay of CaZF. *A*, The 32 bp EP2S sequence tested for gel-shift assay is either wild-type or mutant version M1. Monomers are shown, and tetramers were used in the experiments. Core nucleotides are *underlined* and modified bases are in *bold small case letters*. *B*, Gel-shift assays demonstrating that CaZF binds to the EP2S probe. *C*, CaZF protein localizes in nucleus. Leaf peels of the CaZF overexpressing transgenic and vector transformed plant were analyzed under microscope for phase contrast (a) for GFP activity (c). The sample was restained with DAPI to confirm the nucleus position (b) as indicated. *D*, *E*, Transactivation assay of CaZF in yeast. Full length and truncated CaZF cDNA were cloned into pGBKT7 for expression of CaZF protein as a fusion with GAL4-DNA binding domain. Activation of *HIS3* and *ADE2* reporter genes is shown by growth of the transformants growing in SD (-histidine,-adenine) medium against control (con.) and vector (vec.) transformed (*D*). *LacZ* activation by different deletion constructs of CaZF is shown by β-galactosidase assay (taken as an average of three independent experiments) of the transformants presented as fold increase in activity (*E*).

CaZF possesses EPF type C2H2 zinc finger motifs that has been identified in some transcription factors from petunia by their ability to bind a target sequence EP1S core sequence (TGACAGTGTCA) present in the promoter of their target gene *EPSPS* (5-enolpyruvylshikimate-3-phosphate synthase) [41], [44]. Therefore, CaZF protein was tested for its ability to bind EP1S sequence. EP1S is a 13 bp sequence with an inverted repeat of TGACA separated by a G (Figure 3A). EPF family proteins have spacers of variable lengths between two zinc fingers. Proteins with spacers shorter than 44 amino acids show high specificity of binding to tandemly repeated EP1S with the core G residue separated by 13 bp [44]. Therefore, an EP2S (EP1S dimer) tetramer with 13 bp separations between the core G residues was used as a probe for gel shift assay. Figure 3B shows that glutathion-S-transferase (GST) fused CaZF protein expressed in *E. coli* efficiently bound EP2S tetramer. Expression of CaZF protein fused to green fluorescence protein (GFP) at the C-terminal end under *35S CamV* promoter in tobacco demonstrated that the protein is localized in nucleus (Figure 3C). To determine whether CaZF protein is capable of regulating transcription, *CaZF* ORF was expressed as a fusion to GAL4 DNA-binding domain in a yeast reporter strain carrying *His3*, *Ade2 and LacZ* reporter genes under *GAL4* promoter. Transformed yeast colonies grew on auxotropic medium lacking histidine and adenine (Figure 3D) suggesting that CaZF can function as a transcriptional activator. In order to identify the transactivation domain, two CaZF deletion constructs were introduced into the yeast reporter strain and β-galactosidase activity was assayed. Deletion of C-terminal amino acids after the second C2H2 domain (-Asn) produced higher β-galactosidase activity than the full-length protein (Figure 3E). Increase in transactivation activity after C-terminal deletion of CaZF is most likely due to removal of DLN-box mediated repression. Transcription repressor proteins e.g. ERF, STZ and AZF have a conserved DLN-box motif (^L^/_F_DLN^L^/_F_P) at their C-terminus and that was shown to be essential for repressor activity [45], [46]. Removal of N-terminal amino acids up to the first C2H2 domain (-N99) caused significant reduction of β-galactosidase activity demonstrating essentiality of this domain for transactivation. To further locate the transactivation domain, two more N-terminal deletion mutants, one from 1–44 aminoacids (N44) and other 1–75 aminoacids (N75) were constructed. β-galactosidase assay showed that the aminoacids from 44–75 (L-box) are most important for transactivation property of the protein.

### CaZF-expressing transgenic tobacco plants show salt-tolerance

**Figure 4:**
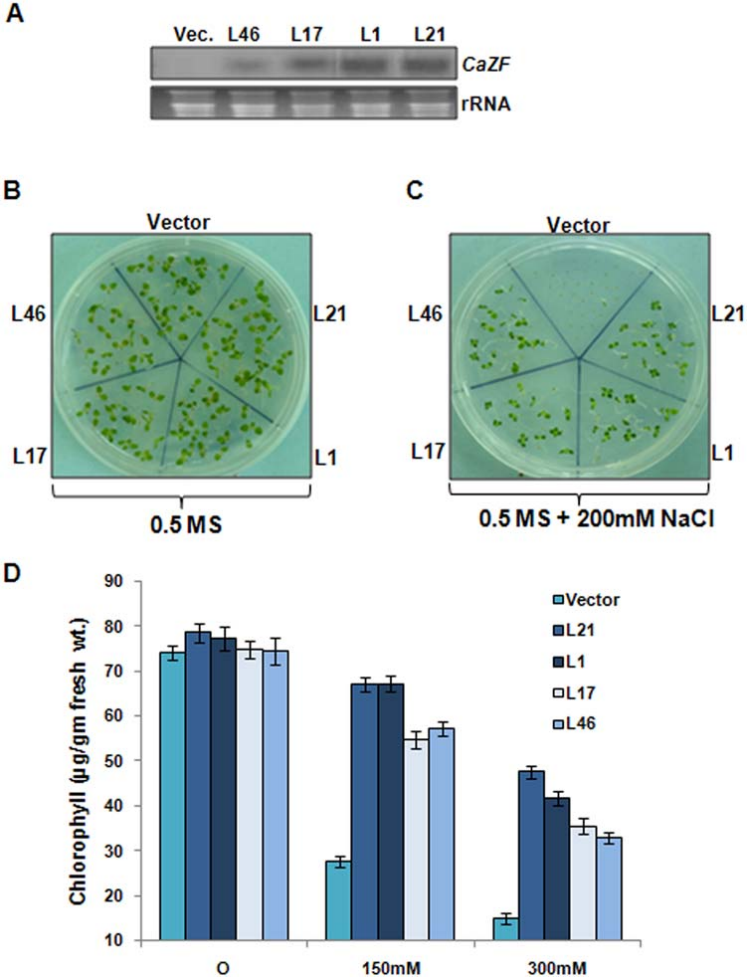
Expression of *CaZF* and salt tolerance of transgenic tobacco. *A*, Northern blot showing expression of *CaZF* in T_1_ transgenic tobacco lines transformed with pBI121 without (vector) or with *CaZF* (L21, L1, L17 and L46). Full length *CaZF* cDNA was used as probe. *B*, Vector control and T_1_ transgenic progenies were grown on 0.5 MS (*Murashige* and *Skoog*) medium for 10 d. *C*, Effect of salt stress on tobacco seedlings from vector control and T_1_ transgenic progenies (CaZFL21, CaZFL1, CaZFL17 and CaZFL46) was demonstrated by germinating seeds on 0.5 MS medium supplemented with 200 mM NaCl for 20 d. Representative figure of three independent experiments are shown. *D*, Chlorophyll content determined by leaf disc senescence assay for salinity tolerance of 30d-old vector control and transgenic tobacco lines overexpressing CaZF, after incubation in water, 150 mM and 300 mM NaCl solutions for 72 hr under continuous white light at 25±2°C. Results of three independent experiments are shown.

To establish the functional significance of CaZF *in planta* the complete ORF of CaZF gene was introduced into tobacco plants using *Agrobacterium*-mediated transformation. Out of twelve transgenic lines harboring single copy of the transgene, two relatively high expressing and two relatively low expressing lines (Figure 4A) were chosen for salt-tolerance analysis. The vector transformed and the CaZF-expressing T_1_ transgenic lines were germinated simultaneously and grew normally in 0.5 MS (*Murashige* and *Skoog*) medium (Figure 4B). To assess the effect of high salt on the seed germination/growth of the vector control and T_1_ plants overexpressing CaZF, surface-sterilized seeds were plated on 0.5 MS supplemented with 200 mM NaCl. In the presence of high salt, vector transformed seeds showed almost no germination (only one out of thirty-two seeds in one repeat) until 20 d, while on average 85–95% CaZF overexpressing T_1_ seeds showed germination within 15 d (Figure 4C). To test for salinity tolerance, leaf disks from all four lines of T_1_ transgenic plants and vector transformed plants were floated separately on water, 150 mM or 300 mM NaCl for 72 h and subsequently total chlorophyll content was quantitated. Chlorophyll content of the vector-transformed and CaZF-expressing plants was comparable in presence of water. However, salinity-induced loss of chlorophyll was much lower in CaZF overexpressing lines (average 13.2% and 23.1% for L1/L21, and 27.8% and 51.2% for L17/L46 at 150 mM and 300 mM NaCl respectively) compared with that in the vector control (average 62.3% and 76.4% at 150 mM and 300 mM NaCl respectively) (Figure 4D). From the damage caused by salt stress it was evident that CaZF overexpressing transgenic tobacco plants have a better ability to tolerate salinity stress as compared to vector control plants. The degree of bleaching (yellow color) observed in leaf disks after 72 h can reflect the extent of damage caused by stress. CaZF-expressing transgenic tobacco seedlings also exhibited improved drought tolerance (data not shown).

### CaZF enhances osmotolerance in yeast

**Figure 5:**
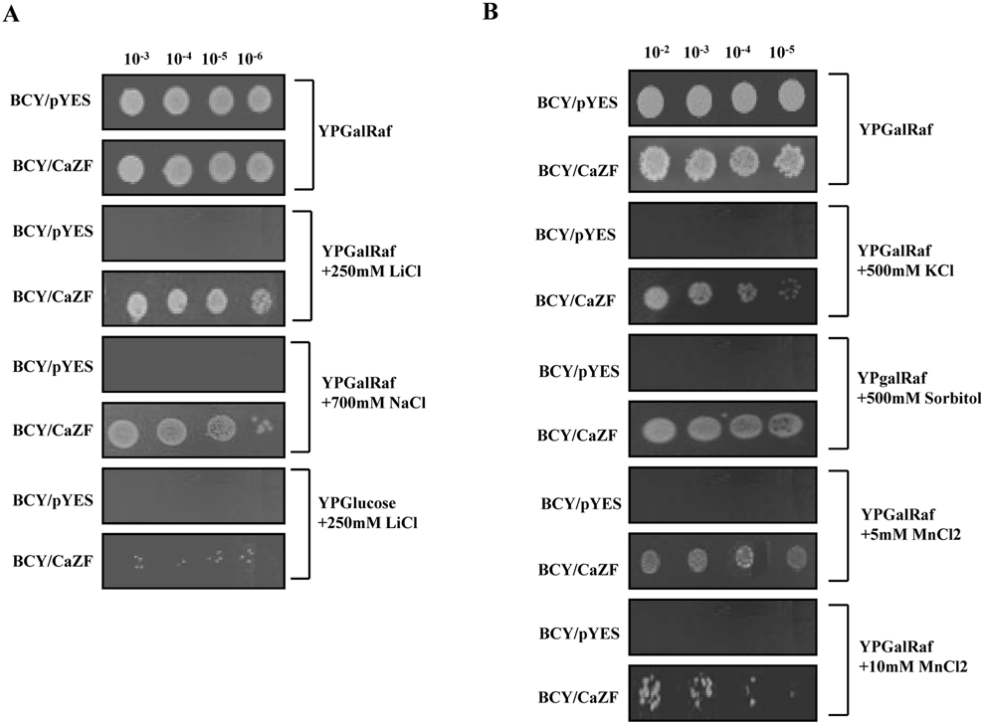
*CaZF* provides tolerance to yeast cells against osmotic stress. *A*, Yeast strain BCY123 harboring only vector (pYES2.1) or CaZF was spotted onto YPGalRaf medium supplemented with 250 mM LiCl or 700 mM NaCl, or onto YPGlu medium containing 250 mM LiCl. Plates without or with salt were shown after incubation at 30°C for 2 d or 4 d respectively. *B*, The same strains, as in *A*, were spotted onto YPGalRaf medium containing either 500 mM KCl, 500 mM sorbitol or MnCl_2_ and incubated at 30°C for 4 d. Representative figures from three independent experiments are shown.

Several reports describe use of yeast mutants to screen and characterize plant salt tolerance determinants [45], [47]. As *CaZF* encodes a ubiquitous C2H2 zinc finger protein, and as yeast and plant stress tolerance systems share quite similar pathways we intended to use yeast salt-sensitive mutants for the characterization of *CaZF*. *CaZF* cDNA was expressed under a galactose-inducible promoter in a protease deficient *S. cerevisiae* strain BCY123 that reduces degradation of the heterologous protein [48]. In contrast to vector transformed control the *CaZF*-transformed colonies were able to grow in galactose-containing medium supplemented with 250 mM lithium chloride (LiCl) or 500 mM NaCl. Li^+^ and Na^+^ are transported through the plasma membrane by same system. The maximum concentration of each salt tolerated by the transformed colonies is 300 mM LiCl and 700 mM NaCl at 10^−6^ dilution when incubated at 30°C for 4 days. Introduction of *CaZF* cDNA in two other yeast strains, BY4742 and PJ69-4A (data not shown) with different genetic background allowed the transformed colonies to grow on a medium supplemented with 250 mM LiCl or 500 mM NaCl, demonstrated that CaZF could function in a broad genetic spectrum. Salt tolerance of the transformed colonies was galactose inducible, as they did not grow LiCl-supplemented medium when galactose was replaced with glucose showing expression of the cDNA was necessary for salt tolerance (Figure 5A). BCY123 harboring *CaZF* cDNA also exhibited tolerance against other ionic and non-ionic osmolytes such as MnCl_2_, KCl and sorbitol (Figure 5B) demonstrating *CaZF* can provide tolerance against general osmotic stress. In liquid medium (YPGalRaf) BCY123 harboring *CaZF* grew almost two fold (doubling time 4.8±0.2 hrs) faster than the vector control strain (doubling time 8.9±0.2 hrs) in presence of 500 mM NaCl. In absence of salt no difference was observed between the growth rates of yeast strains with or without CaZF (doubling time 3.1±0.1 hr) as shown in the solid medium indicating CaZF requires osmotic stress for its function and provides growth advantages only in osmotic stress.

### CaZF partially suppresses salt sensitivity of Calcineurin and HOG pathway mutants

**Figure 6:**
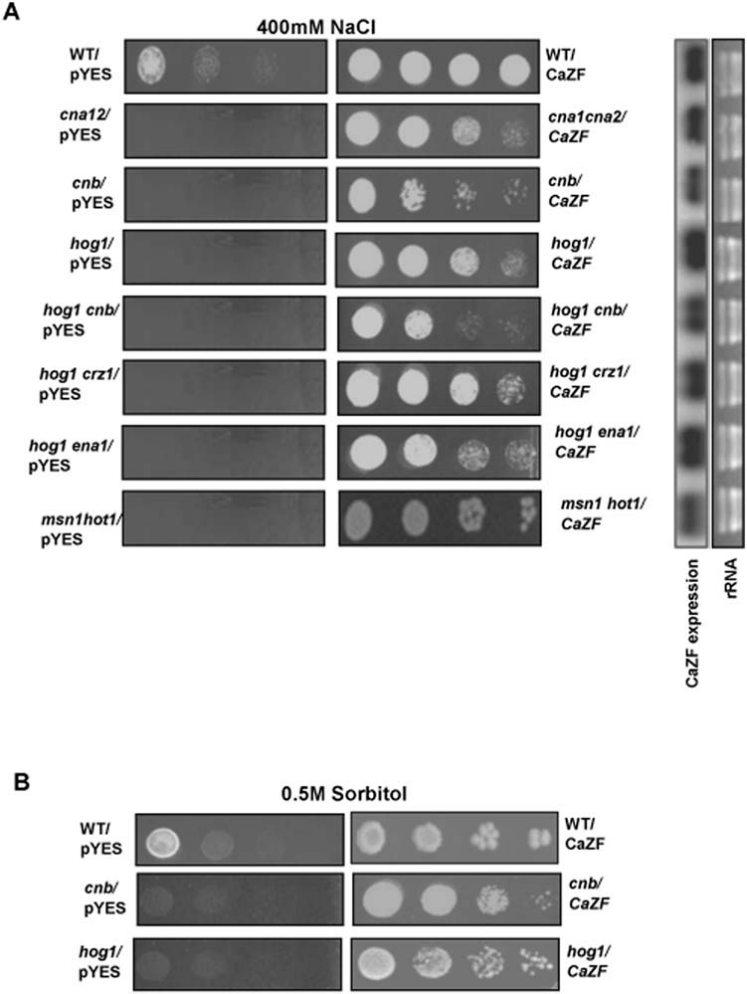
CaZF suppresses salt sensitive phenotype of Hog and Calcineurin mutants. *A*, Wild type and mutant BCY123 cells harbouring either only vector (pYES2.1) or CaZF were spotted onto YPGalRaf plates, containing 0.4 M NaCl. Right panel demonstrated the CaZF expression level in WT and mutant BCY123 yeast strain. rRNA was taken as loading control. *B*, Wild type BCY123 and *hog1* or *cnb* mutants expressing CaZF were spotted onto YPGalRaf medium containing 0.5 M sorbitol. All spotting experiments were performed, as described in Figure. 4. Representative figures from three independent experiments are shown.

Exposure to high salinity causes hypercationic and hyperosmotic stresses to eukaryotic cells [31]. Inter-connected pathways regulated by Hog1p MAP kinase and CAN protein phosphatase determine most of the responses to hyperosmotic stress. A number of salt tolerance determinants in plants have been isolated by their ability to suppress salt sensitivity of the yeast mutants [49]. Therefore, CaZF was tested for its ability to provide osmotic tolerance to some of the well-studied yeast mutants. CaZF suppressed the salt sensitivity when expressed in *cna1cna2* double mutant, lacking both the redundant catalytic subunits and *cnb* mutant lacking the regulatory subunit of Calcineurin on 400 mM NaCl (Figure 6A). However, CaZF could not protect the *cnb* mutant against the ionic osmolytes KCl (data not shown) and NaCl to the extent as it did for the wild type cells. This was also reflected when growth rates were measured in liquid medium. *cnb* mutant cells expressing CaZF grew faster (doubling time 7.2±0.16 h) than that carrying only vector (doubling time 11.8±0.3 h) but grew at a much slower rate than the wild type cells expressing CaZF (doubling time 4.8±0.2 h). These data indicate that CaZF functions through a pathway, which is additive to but independent of Calcineurin pathway. Alternatively, CaZF can partially complement salt sensitivity of the *can* mutants but requires the CAN pathway for its full function. Interestingly, growth rates of the *cnb* mutant and the wild type cells harboring CaZF in non-ionic osmolyte sorbitol containing medium were comparable (Figure 6B). The probable explanation is Calcineurin pathway protects the cells against toxicity of only ionic osmolytes while HOG pathway protects against hypertonic stress due to both ionic and nonionic osmolytes [50].

As CaZF enhanced growth of wild type yeast in presence of nonionic osmolyte, we wanted to test whether it can function in the background of HOG pathway mutants. Expression of *CaZF* suppressed salt sensitivity of *hog1* mutant. As in case of the calcineurin mutants, CaZF provided much less tolerance to the *hog1* strain than it provided for the wild type strain carrying only vector against the ionic and nonionic osmolytes. The result in the solid medium was also supported by the growth rates in liquid medium with 500 mM NaCl; as doubling time of *hog1* strain carrying pYES was 12.0±0.15 h as opposed to 7.8±0.2 h for the *hog1* cells expressing CaZF that was much higher than that required for the wild type cells expressing CaZF (4.8±0.2 h). The osmosensitive phenotype of yeast mutant lacking both *Hot1* and *Msn1*, transcriptional activators of the Hog pathway, can also be suppressed by *CaZF* expression (Figure 6A) in solid and liquid hypertonic medium as well. Under hypertonic stress Hog1p, through Sko1p, activates expression of *Ena1* [28], [51], [52], which is also regulated by Calcineurin independent of Hog1p through a transcription activator Crz1p, a C2H2 zinc finger protein [3], [4], [12]. As CaZF could suppress osmosensitivity of *hog1* and *cnb* mutants separately, we constructed a double mutant *hog1cnb* to test the functional ability of *CaZF* in absence of both Hog1p and Calcineurin. Surprisingly, *CaZF* expression partially suppressed salt sensitivity of *hog1cnb*. Analysis of the effect of CaZF expression in these mutants of HOG and CAN pathways mutants suggests that CaZF mediated suppression of osmosensitivity involves a pathway(s) that is independent of but additive to Hog1p and Calcineurin regulated pathways. Simultaneously, reduced growth rate of CaZF expressing mutant cells in comparison to the wild type cells expressing the plant gene also evokes a possibility that CaZF may require both the pathways to function in its full strength. The data presented above suggests that hyperosmotic-adaptation pathway (s) independent of Hog1p and Calcineurin can be created in yeast.

### CaZF induces expression of HOG and Calcineurin regulated genes

**Figure 7:**
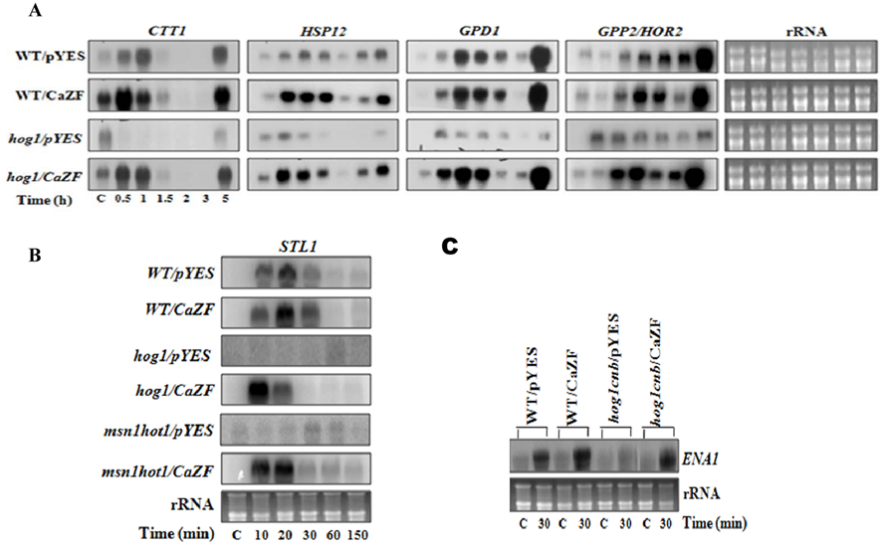
Effect of CaZF on stress-responsive gene expression. Wild type or mutant cells as mentioned harboring either only vector (pYES2.1) or CaZF were grown in YPGalRaf medium and treated with 500 mM NaCl for mentioned period of time. Northern analysis was performed with probes representing genes mentioned in the figure subpanels *A*, *B* and *C*. Representative figures from three independent experiments are shown.

We have shown that CaZF can enhance hyperosmotic stress tolerance in budding yeast and it can partially suppress the osmosensitive phenotype of the mutants lacking Hog1p and/or Calcineurin activities. We further investigated whether *CaZF* expression has any influence on expressions of the genes those are regulated by HOG1p and involved in glycerol production and damage repair. We analyzed in wild type and *hog1* background, expressions of two genes, *GPD1* and *GPP2*, involved in glycerol synthesis and two general stress response genes *CTT1* and *HSP12*, predominantly controlled through STRE. Expressions of all the four genes, with different expression kinetics, in the vector control wild type are enhanced quickly after the salt stress and remained expressed even after 3 h (Figure 7A). Expression of CaZF did not show any significant effect on the expression of these genes under control condition except an increase in *CTT1* expression. However, under salt stress, expression of *CTT1* and of *HSP12* throughout the course of experiment was higher in CaZF expressing cells, suggesting that CaZF function in yeast is not constitutive and it requires some stress-induced pathway(s) for its function. As expected, *hog1* mutation caused significant reduction in expression of all these genes. Surprisingly, *hog1* mutant harboring CaZF induced expression of all these genes almost at the level of CaZF expressing wild type strain in response to salt stress. HOG pathway regulated genes were shown to recruit Hog1p at their promoter for the osmotic stress-mediated expression. Salt induced expression of these genes in absence of Hog1p suggests that either CaZF along with some Hog1p-independent factors is directly activating the expression of these genes or a Hog1p-independent salt-inducible pathway in yeast is activated by CaZF under salt stress and ultimately causing expression of these genes. CaZF-regulated Hog1p-independent expression of these genes though is not sufficient for providing osmotolerance to the extent as with the wild type background indicating that Hog1p is indispensable for a part of the osmoadaptation mechanism.

Expressions of the genes mentioned so far are also regulated by complex mechanisms, which are not related with HOG pathway and are also involved in other stress responses [53]. Therefore, we have analyzed a comparatively simpler expression system that is exclusively controlled by Hog1p. As reported earlier [48], *STL1* (a gene encoding a putative hexose transporter) expression is completely abolished in *hog1* and *hot1* mutants in response to salt stress. Hog1p and Hot1p are recruited in an interdependent manner on *STL1* promoter during acute salt stress as supported by chromatin immunoprecipitation [54]. In this study, expression of *STL1* was undetectable in absence of salt stress in wild type cells with or without CaZF expression, again supporting the data that the plant protein is not constitutively active in yeast. In presence of 500 mM NaCl, *STL1* expression was quickly increased with a peak at 20 min at the experimental condition (Figure 7B). Expression of CaZF had no significant effect on *STL1* expression in wild type cells in presence of salt. As expected *STL1* expression was undetectable in *hog1* mutant, however, under salt stress CaZF in *hog1* mutant not only induced *STL1* expression to the extent as it did in wild type background, the expression peak was shifted to 10 min; the shifting of peak of *STL1* expression was also evident in *msn1hot1* double mutant, where the expression at 10 min was comparable to that at 20 min. Interestingly, CaZF-mediated *STL1* expression persisted for relatively shorter time period in *hog1*. Induction of *STL1* expression in response to salt stress in *hog1* and *msn11hot1* mutants confirmed that CaZF functions in a salt stress-dependent manner in yeast and indicates that CaZF also possesses a Hog1p-independent function to activate the expression of some Hog1p-regulated genes.

As CaZF could partially suppress salt sensitivity of *hog1cnb* double mutant we intended to analyze *ENA1* expression, which is regulated independently by both Calcineurin and Hog1p, in CaZF expressing cells (Figure 7C). In wild type and mutant background, CaZF did not alter steady state low expression level of ENA1 transcript in control condition. Expression of *ENA1* was increased rapidly after exposure to salt in wild type cells and that is further enhanced by more than 1.5 fold in cells harboring CaZF. *ENA1* expression was hardly detectable in *hog1cnb* cells and that is slightly enhanced in response to salt. However, in CaZF expressing mutant cells *ENA1* transcript was accumulated at an equivalent level of wild type cells. To test whether *ENA1* gene product is essential for CaZF function, it was expressed in *hog1ena1* double mutant. Figure 6A shows that CaZF expression suppressed salt stress sensitivity not only of *hog1cnb* but also of *hog1crz1* and *hog1ena1* double mutant indicating ENA1 is not essential for CaZF function.

Both HOG and CAN pathways function through a number of transcriptional activators of zinc finger family of proteins e.g. Msn2p/Msn4p, Sgd1p and Crz1p/Tcn1p. Among them Msn2p/Msn4p and Crz1p belongs to C2H2 zinc finger family [3], [4]. Despite of the fact that Msn2p/Msn4p and Crz1p are much larger proteins and have no overall sequence similarity with CaZF, the plant protein most likely is able to functionally substitute both these transcription activators. This is quite evident in case of *CTT1*, *HSP12* and *ENA1* expression in CaZF expressing wild type cells in stress. Expressions of *CTT1* and *HSP12*, predominantly regulated by Msn2p/Msn4p through STRE [21], [55] and that of *GPD1* and *GPP2*, regulated by Hot1p and Msn1p [14], [19] and not dependent on functional STREs, reveals a striking difference when compared in CaZF-expressing wild type cells in presence of salt. In wild type cells CaZF further induced expressions of *CTT1* and *HSP12* but not of *GPD1* and *GPP2* in salt stress. Thus, CaZF function is not redundant rather additive to Hog1p-mediated expression of these C2H2 zinc finger and STRE-regulated genes. In *hog1* background, under salt stress CaZF is able to induce expression of these genes to a similar extent as Hog1p does in the wild type cells. This also seems to be the mechanism for Crz1p-regulated gene *ENA1*. *ENA1* expression is partially regulated by Crz1p [4], [28]. Accordingly, CaZF further enhanced *ENA1* expression by only about 1.5 fold in wild type background under salt stress. In *hog1cnb* background it was found that CaZF was able to activate expression of *ENA1* independent of Hog1p and Calcineurin to the same level as those regulatory enzymes do in wild type cells. While Crz1p requires Calcineurin-mediated post-translational modification to be active [3], CaZF does not require that. However, CaZF seems to require Hog1p and Calcineurin-independent but stress-dependent post-translational modifications and/or protein-protein interaction for its full functional ability.

For the genes (*GPD1*, *GPP2* and *STL1*) that are regulated predominantly by Hot1p, which is not a C2H2 zinc finger protein, influence of CaZF on their expression was evident only in absence of *Hog1* and *Hot1*. *GPD1* and *GPP2* expressions are also regulated by other proteins (e.g. Rap1p for *GPD1*) [56] and irrespective of combination of gene knockouts involving Hog1p and Hot1p; *GPD1* and *GPP2* remains salt inducible. But CaZF seems to induce expression of these genes by similar mechanism used by Hog1p and Hot1p. The reason being it induces expression of *STL1*, which is exclusively regulated by Hog1p and Hot1p [54]. There is possibility that CaZF utilizes other proteins and/or other salt inducible pathways to mimic Hog1p-regulated activation, but cannot totally replace Hog1p as *STL1* expression kinetics differs in presence and in absence of Hog1p. CaZF seems to require these genes for salt-tolerance because their end product, the glycerol synthesis in the mutant cells harboring the plant gene nicely correlates their growth in presence of salt.

ENA1, a P-type ATPase, is the first member of cluster of four to five genes encoding very similar proteins and plays a major role in detoxification of sodium and lithium cations. A complex mechanism involving different pathways regulates *ENA1* induction in response to salt. Involvements of Hog1p and calcineurin are already discussed. At least two other pathways namely TOR and Hal3/Ppz also regulate *ENA1* expression in response to salt [for review [57], [58]]. However, in our experimental system *ENA1* expression increased marginally in *hog1cnb* cells in response to salt suggesting these two enzymes (Hog1p and Calcineurin) are the major regulators of salt-responsive *ENA1* expression. Expression of CaZF confers salt tolerance and induces ENA1 expression in *hog1cnb* suggests that CaZF functions by activating Na^+^/Li^+^ extrusion system and at the same time also by mechanisms not involving ENA1p as it enhanced salt tolerance of *hog1ena1* mutants. *ENA1*-independent mechanism may involve other cation efflux systems, such as *NHA1* and *SNQ2* or K^+^-influx systems like *TRK1* [7], [59], [60]. Similar *ENA1*-independent salt tolerance was also provided by other plant proteins e.g. STO and SLT1 [61], [62].

### CaZF induces glycerol accumulation

**Figure 8:**
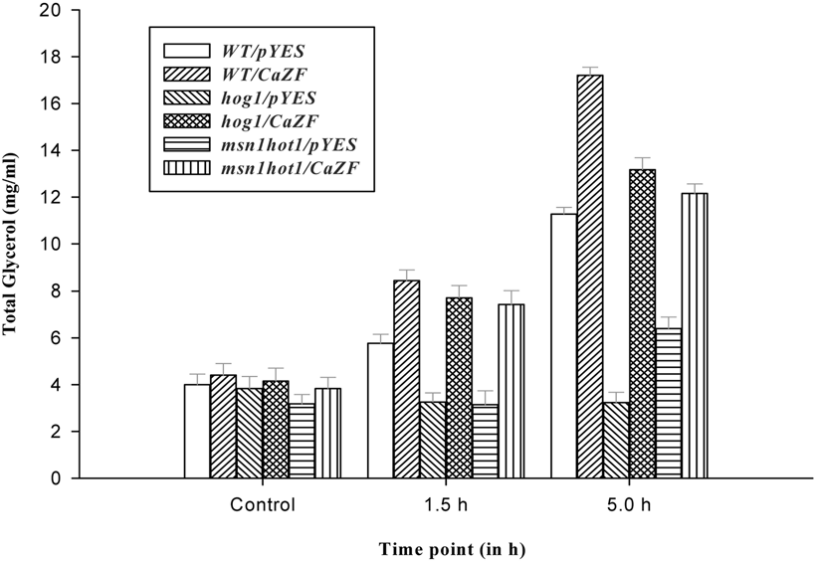
Effect of CaZF expression on accumulation of glycerol in response to salt treatment. Total glycerol [(mg/ml); equivalent to Triloen content] was estimated in wild type and mutant cells carrying either only vector (pYES2.1) or expressing CaZF after treatment with sodium chloride for mentioned time period in the figure. Glycerol assay was done with three experimental repeats and the average value was considered.

To further clarify, whether the effects of CaZF on HOG and CAN-pathway gene expression described above are relevant for a functional osmotic stress response; we estimated glycerol production during or after stress exposure. Figure 8 shows that in control condition mutation of the HOG pathway genes and *CaZF* expression had no influence on constitutive level of total glycerol. Mutant lacking *Hog1* did not stimulate glycerol production in response to stress even at the later stage, while *msn1hot1* double mutant started accumulating glycerol later in stress. However, irrespective of genetic background CaZF enhanced production of glycerol in response to stress though comparatively less in the mutant strains than in the wild type cells corroborating the comparative osmotic tolerance levels of the wild type and the mutant strains expressing CaZF. This result shows that CaZF not only increases the expression of HOG-pathway genes, but also activates the functional osmotic stress response to salt stress in absence of *Hog1*.

### CaZF directly binds to STRE and activates CTT1 promoter

**Figure 9:**
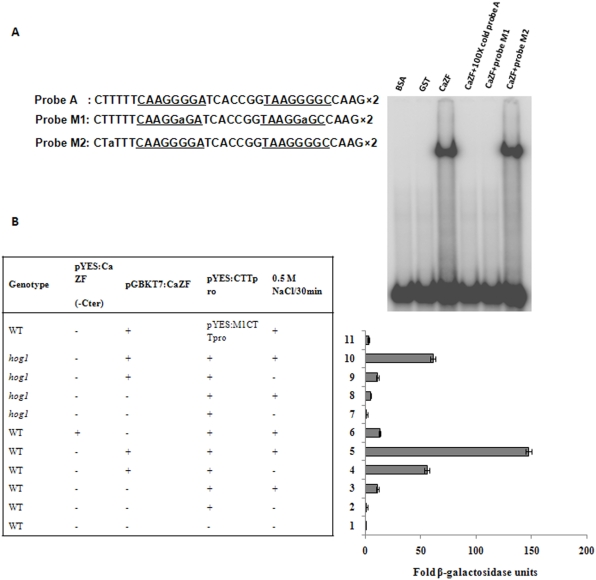
CaZF directly binds to STRE and activates CTT1 promoter. *A*, Gel-shift assay demonstrating that CaZF binds to the STRE sequence containing probe. Either wild-type or mutant versions (M1 and M2) are used. STRE sequences are *underlined* and modified bases are in *bold small case letters* (left panel). *B*, Transactivation assay of CTT1-*LacZ* construct by CaZF. Full length or truncated CaZF protein and LacZ reporter fused to CTT1 promoter fragment or its mutant were cointroduced in wild type (WT) or *hog1* BCY123 yeast strains. The transformed yeast strains were treated with/without 500 mM NaCl for 30 min. Activity of β-galactosidase of each sample (average of three independent transformants) as mentioned in table (left panel) was determined and presented in the form of fold induction in activity (right panel).

*CTT1*, *HSP12*, *GPD1* and *GPP2*, the well-studied general stress response genes regulated by HOG pathway possess stress tolerance responsive elements in their upstream activating sequence. Msn2p and Msn4p, two C2H2 zinc finger proteins bind to STRE of *CTT1* and *HSP12* to activate their expressions. To determine the mechanism of action of *CaZF* in yeast we, therefore, tested the ability of CaZF to bind STRE. CaZF protein fused to glutathione-S-transferase (GST) was purified from bacteria and used for gel mobility shift assay using a radiolabeled probe derived from *CTT1* promoter having tetramer of STRE core element (AAGGGG). Figure 9A clearly shows that CaZF bound to STRE in a sequence specific manner as replacement of a ‘G’ residue with ‘A’ residue in the core element of the probe (M1) totally abolished the binding while another replacement outside the core element (M2) maintained the binding efficiency.

We have shown that expression of CaZF was able to induce expression of *CTT1* (and three other STRE-containing genes) in *hog1* background. Therefore, we tested the ability of CaZF to activate *CTT1* promoter in wild type and *hog1* mutant. 800 bp (−137 to −937) upstream activating sequence including the translation start codon of *CTT1* was amplified and inserted before *LacZ* reporter gene to regulate its expression. Wild type and *hog1* yeast strains were co-transformed with the reporter construct and CaZF-expressing plasmid and activity of LacZ was assayed. Figure 9B shows that CaZF induced *CTT1* expression by more than 50 fold in absence of salt and almost 150 fold of the basal level in presence of salt in the wild type strain. In *hog1* background the inductions were 10 and 60 folds in absence and in presence of salt stress respectively. A CaZF deletion construct lacking its transactivation domain could not activate the *CTT1* promoter and a *CTT1* promoter construct with mutated STRE was not activated by CaZF. This result demonstrated that CaZF was able to activate yeast stress tolerance responsive element in Hog1-independent manner by directly binding to it.

### Requirement of CaZF C-terminal domain for salt tolerance

**Figure 10:**
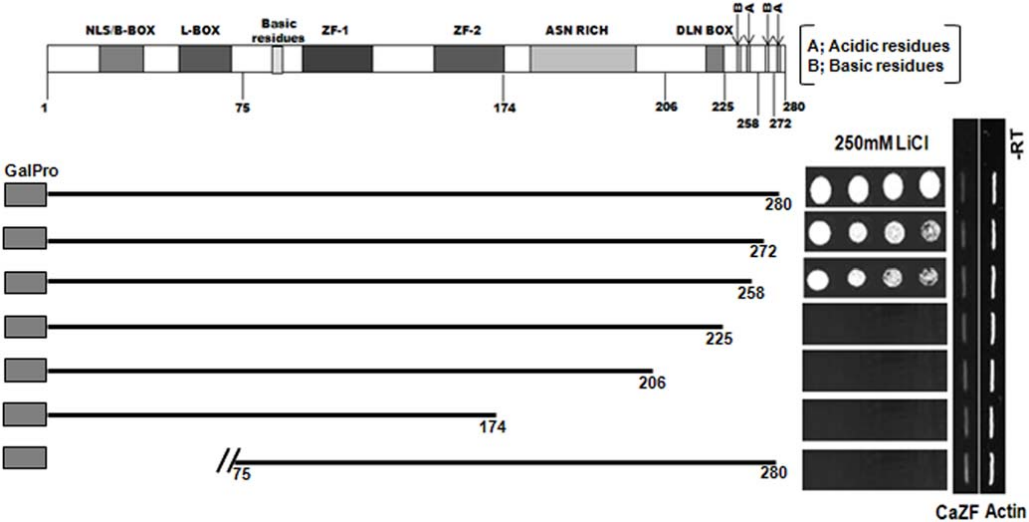
Determination of salt tolerance ability of *CaZF* deletion constructs. BCY123 cells transformed with C-terminal deletion constructs of CaZF as shown in the schematic representation were tested for their ability of providing salt tolerance against 250 mM LiCl. Representative figures from three independent experiments are shown.

Transactivation assay in yeast determined that the N-terminal domain of CaZF is responsible for its transactivation property and accordingly removal of that domain made CaZF unable to provide salt-tolerance. To investigate whether the C-terminal domain has any role in its activity, we made serial deletion constructs from the C-terminal end of the protein. Removal of last 22 amino acids did not make any difference in the activity of CaZF. However, further removal of 33 amino acids totally abolished the capability of CaZF to provide any salt-tolerance (Figure 10). A close analysis of the amino acid sequence reveals that this domain contains apart from alternate stretches of basic and acidic amino acids, a potential site for protein kinase C phosphorylation (SKK) and a potential site for cAMP/cGMP-dependent protein kinase phosphorylation (KKKS). In yeast, cAMP-dependent protein kinase A (PKA) is an essential component of general stress response pathway. In normal growth condition PKA phosphorylates C2H2 zinc finger proteins Crz1p, Msn2p and Msn4p to prevent their nuclear localization. Upon inactivation of PKA or activation of the phosphatase calcineurin during stress those proteins get dephosphorylated and are accumulated in the nucleus to activate their target genes [63]–[65]. CaZF, being a C2H2 zinc finger protein may be regulated by the same mechanism and, thereby, stress-mediated activation of CaZF-function can be explained. Altogether, these results suggest importance of the C-terminal domain with acidic and basic stretches in CaZF function.

### Conclusion

We have identified a chickpea gene, CaZF encoding a C2H2 zinc finger protein, which is expressed relatively in higher amount in response to drought stress in a drought-tolerant variety in comparison to a sensitive one. We raised tobacco transgenics overexpressing CaZF showing tolerance to high salinity.

Most likely, in yeast, CaZF does not function at the level of Hog1p; rather it works downstream to it. The reasons being, CaZF structurally resembles a transcription factor and expression of *CaZF* is not toxic like expression of ASR1. ASR1 expression in control condition was growth-inhibitory like a constitutively active Hog1p mutant [47], [66]. In spite of inducing gene expression and glycerol production, expression of CaZF could not provide equivalent salt-tolerance to *hog1* and *cnb* mutants as it did in wild type cells. There might be several reasons for that. We have analyzed expressions of only a few genes while Hog1p and Calcineurin together regulate expression of a wide number of genes involved in several pathways. Secondly, Hog1p-mediated stress relief mechanism begins much before of Hog1p-regulated transcriptional induction of downstream genes. Osmotic stress-activated Hog1p phosphorylates Nha1 Na^+^/H^+^ antiporter, which is crucial for rapid reassociation of those proteins, which were dissociated from the chromatin due to stress, with their target sites [24], [67]. Apart from that, HOG pathway shares kinases and phosphatases with a lot of interconnecting pathways [for review [53]], which cannot be replaced totally by heterologous expression of one transcription factor.

In conclusion, expression of *CaZF* in yeast provides evidence that at least some of the crucial stress tolerance determining genes, which are regulated by Hog1p MAP kinase, Calcineurin protein phosphatase and their target transcription factors during osmotic stress, can also be activated to the same extent in absence of their regulatory enzymes/transcriptional activators. Activation of those genes by a heterologous gene leads to production of the HOG pathway end product i.e. glycerol. In at least one previous instance it was shown that a plant gene (*ASR1*) could induce synthesis of glycerol in salt stress in absence of Hog1p [47], [66]. The level of dependence of yeast cells on *Hog1* differs with intensity and extent of stress conditions. After 20 min of exposure at 800 mM NaCl, 75% of salt stress-responsive genes are strongly dependent on *Hog1* while only 32% of them are strongly dependent on *Hog1* after 10 min exposure at 400 mM NaCl exposure. At 400 mM NaCl 36% of salt-induced genes are independent of *Hog1* in contrast to only 3% at 800 mM [2]. Therefore, influence of Hog1p on expression of genes is relative to the experimental condition. We have analyzed gene expression and glycerol estimation at 500 mM NaCl and so there may be a possibility that in this experimental condition influence of Hog1p is relatively less in providing salt-adaptation. A salt-inducible but Hog1p and Calcineurin-independent pathway definitely uses CaZF as a substrate for post-translational modification and/or target for protein-protein interaction, because it can induce gene expression and consequently provide growth advantage only in presence of stress; and secondly it requires its C-terminal domain, which is not required for its transcription activation property, for its function. CaZF might be a potential target for cyclic AMP-dependent protein Kinase such as protein kinase A. Altogether our experiments in tobacco and in yeast demonstrate that CaZF, a C2H2 zinc finger protein from chickpea is a potential salt tolerant determinant in plant. Unlike several other zinc finger proteins having ‘DLNL’ motif and acting as transcription repressor, CaZF acts as transcription activator. Other than its transactivation domain, which resides at its N-terminus, the C-terminal aminoacids also play a major role in its activity. CaZF requires post-translational modification and/or interaction with other stress-inducible proteins for its full activity. Our results in yeast model suggest that CaZF can act as a general osmotolerance-determinant by inducing the production of osmolytes by directly activating their promoters.

## Materials and Methods

### Yeast Strains and Culture Conditions

Yeast strains used in this study are BCY123, BY4742 and PJ69-4A (Table 1). Different mutants used in this study are the derivatives of *Saccharomyces cerevisiae* BCY123 (wild type) [48]. To culture cells, standard yeast media and growth conditions were used. Yeast cells were grown in either YP containing 2% bacto-peptone, 1% Difco yeast extract, 50 µg/mL adenine sulphate supplemented with either 2% dextrose (YPD) or 2% galactose and 2% raffinose (YPGalRaf) or synthetic media containing 0.7% (w/v) yeast nitrogen base supplemented with the required amino acids at 30 µg/mL, 2% (w/v) Glucose, 50 mM succinic acid/Tris (pH 5.5) at 30°C. *Escherichia coli* strain DH5α, used in this study, was grown in Luria broth (LB) medium containing 1% peptone, 0.5% yeast extract and 0.5% NaCl supplemented with ampicillin (50 mg/L) at 37°C. Antibiotics were filter sterilized and added to autoclaved medium.

**Table 1:** *S. cerevisiae* strains used in this study.

S. NO.	STRAIN	GENOTYPE	SOURCE OF REFERENCE
1	BCY123	*Mat a*, *pep4::HIS3 prb1::LEU2 bar1::HISG lys2::GAL1/10-GAL4 can1 ade2 trp1 ura3 his3 leu2-3*, *112 Δlys2cir*+GAL+RAF+SUC	[48]
2	BCY123a	Same as BCY213, except *cna1*::HIS3 *cna2*::TRP1	This Study
3	BCY123b	Same as BCY213, except *cnb*::TRP1	This Study
4	BCY123c	Same as BCY213, except *hog1*::TRP1	This Study
5	BCY123d	Same as BCY213, except *msn2*::HIS3 *msn4*::TRP1	This Study
6	BCY123e	Same as BCY213, except *msn1*::HIS3 *hot1*::TRP1	This Study
7	BCY123f	Same as BCY213, except *hog1*::TRP1 *cnb*::HIS3	This Study
8	BCY123g	Same as BCY213, except *hog1*::TRP1 *crz1*::HIS3	This Study
9	BCY123h	Same as BCY213, except *hog1*::TRP1 *ena1*::HIS3	This Study
10	BY4742	MAT α *his3Δ leu2Δ lys2Δ ura3Δ* YJL059W::kanMX4	[76]
11	PJ69-4A	*MATa trp1-901 leu2-3,112 ura3-52 his3-200 gal4Δ gal80Δ LYS2::GAL-HIS3 GAL2-ADE2 met2::GAL7-lacZ*	[71]

### Plant material used and Identification of cDNA coding for *CaZF*

A drought-tolerant (BGD72) and a drought-sensitive (ICCV2) cultivar of chickpea (*Cicer arietinum*) were used in this study. The cultivars were grown in same pot containing soilrite∶vermiculite (1∶1). The seedlings were grown in same pot to keep same soil moisture content for both the cultivars. Drought treatment was applied by stopping irrigation to 10 d old chickpea seedlings. Samples were harvested after 0, 3, 6 and 12 d post-irrigation. Subtractive cDNA libraries constructed with these cultivars at different stages of drought resulted in some EST clones that express higher in the drought-tolerant cultivar in response to drought. One such EST encoding C2H2 zinc finger protein was used in this study. 5′RACE System (Life Technologies, Rockville, MD) was used to construct the full length cDNA of *CaZF*. For functional study in yeast the complete and truncated ORF of CaZF was directionally cloned into pYES-2.1-V5 His-TOPO flanked by *XhoI* and *XbaI* restriction sites under galactose-inducible GAL1 promoter.

### Subcellular Localization Analysis of Transiently Expressed Fusion Proteins

The CaZF coding region without the translation stop codon was cloned in pCAMBIA1302 to produce the protein fused to GFP using following PCR primers (5′CATGCCATGGCTTTAGAGTTAGAAGCT3′) and (5′GAAGATCTTGCACCGTTTCATCATC3′). The PCR amplified fragments were digested with NcoI and BglII and cloned in pCAMBIA1302 vector. The construct was introduced into tobacco (*Nicotiana tabaccum cv. xanthii*) by *Agrobacterium* mediated transformation. For the nuclear staining, tobacco leaf peals were incubated for 10 min with DAPI (1 µg/µl) before observing under fluorescent microscope with FITC filter.

### Raising of *CaZF* Overexpressing Transgenic Tobacco Plants

The complete ORF of *CaZF* gene was cloned into the *Xbal*-*Smal* site of the pBl-121 vector (Clontech) in the sense orientation. pBI121 without (vector-control) and with *CaZF* were chemically mobilized in to *Agrobacterium tumefaciens* strain GV3101. Tobacco (*Nicotiana tabaccum cv. xanthii*) leaf discs were transformed following standard protocol [68]. Putative T_0_ transgenic plants were regenerated from the callus in the presence of kanamycin and integration of the transgene was further confirmed by PCR amplification. The seeds from these plants, i.e. T_0_ seeds, were germinated on kanamycin-containing medium and on the basis of segregation analysis and genomic Southern blot; transgenic lines with single transgene insertion were selected for further analyses.

### Leaf Disc Assay of CaZF Transgenic Plants

Leaf discs of 1.0 cm diameter were excised from healthy and fully expanded tobacco leaves of same age (30 d post germination) from *CaZF*-expressing and vector-control plants. The discs were floated in a solution of NaCl (150 mM or 300 mM) or water (experimental control) for 72 h [69]. The discs were then used for measuring chlorophyll spectrophotometrically after extraction in 80% cold acetone. The salinity and water treatments were carried out in continous white light at 25±2°C. The experiments were done with three experimental repeats of each vector-control and transgenic lines.

### Preparation of Recombinant Proteins and Gel Mobility Shift Assay

To generate a GST-fusion protein, the corresponding ORF of CaZF was amplified by PCR with primers flanked with restriction site for *EcoRI* and inserted into pGEX4T2 expression vector and introduced into *Escherichia coli* BL21 (DE3). Protein expression was induced by 0.5 mM IPTG for 3 h at 30°C. The recombinant proteins were purified from bacterial lysates with Glutathione-Sepharose beads (GE-Amersham) and subsequently monitored by 10% SDS-PAGE. All DNA binding reactions were carried out in 25 mM HEPES-KOH; pH 7.6, 40 mM KCl, 0.1% Nonidet P-40, 0.01 mM ZnCl_2_, 10 µg/ml poly (dI-dC), and 0.1 mM dithiothreitol. Gel-shift assays were performed with 10,000 c.p.m. of ^32^P-end-labeled probe A, a tetramer of TTGACAGTGTCACGCG TTGACAGTGTCACGCG (core nucleotides are underlined) or mutated probe M1, a tetramer of TT**c**A**g** AGTGTCAC**c**C**g**TTGACAGTGTCACGCG (mutated bases are in bold lower case letters. After incubation for 20 min at room temperature, the mixtures were subjected to electrophoresis in 8% polyacrylamide gel as described previously [70].

### Yeast One-Hybrid Assay

CaZF protein coding sequence or the truncated forms were cloned in yeast (*S. cerevisiae*) expression vector pGBKT7 (Clonetech) at *NdeI-EcoRI* site to express CaZF proteins fused to GAL4 DNA-binding domain. The constructs were transformed into an auxotropic yeast strain PJ69-4A [71] that contains three reporter genes, *HIS3*, *ADE2*, *and β-GAL*, under the control of GAL4 promoter, and plated on synthetic medium lacking histidine and adenine. β-Galactosidase assay of three independent transformed colonies was done in triplicates with ortho-nitrophenyl-β-D-galactoside (ONPG). Presence of different form of CaZF in the transformed colonies were confirmed by PCR and sequencing.

### Gene disruption, Complementation and Transformation of Yeast

Direct gene deletion of the target genes with the marker module was done by PCR-based gene deletion strategy [72], [73] using the primers mentioned in Supplemental Table S1. *HIS3* and *TRP1* markers were amplified from plasmids pRS413 and pGBKT7 respectively. Correct deletion of the target genes was detected by diagnostic PCR using whole yeast cells from isolated colonies and a set of oligonucleotides designed to bind outside or inside of the replaced target sequence and within the marker module. Disruption of Hog1 gene was confirmed by Western Blot analysis with Hog1p-specific antibody (Supplemental Figure S1). After confirmation of the fidelity of the constructs by sequencing, different yeast strains were transformed with constructed plasmids or with empty pYES-2.1 by the Lithium-acetate/PEG method [74]. Transformants were selected for uracil prototrophy by plating on synthetic media lacking uracil (SC-Ura^−^). Ura^+^ colonies were selected thereupon. For osmotolerance experiments and to monitor the growth of mutant yeast strains complemented by CaZF and/or truncated forms, drop tests were performed.

### Yeast Spot Assay

For drop tests, overnight YPGal grown yeast cells were diluted to OD_600_ = 0.4 in 2% Gal, 50 mM MES pH 5.5 and incubated for 3 h and then further serially diluted with YP to obtain 10, 10^2^, 10^3^ and 10^4^ cells. Three microliters of each dilution was then spotted onto YPGalRaf with/without NaCl, LiCl, KCl or sorbitol as mentioned or onto complete synthetic uracil^−^ medium supplemented with 2% Gal, 0.2% sucrose and MnCl_2_ as indicated in the figures. Plates were incubated at 30°C and unless otherwise indicated, colony growth was inspected after 2–4 d.

### RNA Isolation

Cells were grown in YPGalRaf at 30°C to late log/stationary phase. Cultures were diluted to an OD_600_ of ca. 0.1 in YPGalRaf medium, and then further grown at 30°C till OD_600_ reached to 0.5. Then the, cells were subjected to saline stress for different time points as mentioned in figures. The saline stress was given by suspending pelleted cells in salt-containing medium. After saline stress, cells were centrifuged for 3 min at 7,000 rpm, and total RNA was extracted from untreated cells or cells treated with NaCl by using hot phenol method as described [75].

### Northern and Western Blot Analyses

For Northern blot analysis, total RNA (20 µg/lane) was electrophoresed on 1.2% agarose-formaldehyde gels and transferred to positively charged nylon membranes (GE-Amersham, UK). Membranes were hybridized at 60°C in the presence of hybridization buffer (700 mM NaCl, 40 mM NaH_2_PO_4_;pH 7.6, 4 mM EDTA, 0.2% polyvinylpyrrolidone, 0.2% Ficoll, 0.1% SDS, 0.2 mg/ml salmon sperm DNA) and 10^6^ cpm/ml appropriate ^32^P-labeled DNA fragment. DNA fragments containing the ORF of the following genes were used as probes: *CTT1* (YGR088W) from position +1 to +540 (0.54 kb), *HSP12* (YFL014W) from position +1 to +330 (0.33 kb), *GPD1*(YDL022W from position +1 to +540 (0.54 kb), *GPP2/HOR2* (YER062C) from position +1 to +480 (0.48 kb), *ENA1* from position +90 to +1000 (0.91 kb) and *STL1* (YDR536W) from position +40 to +1032 (0.99 kb). Probes were labeled using the random-primed DNA labeling kit (GE-Amersham, UK). Filters were washed in 0.1× SSC (1× SSC is 150 mM NaCl, 15 mM sodium citrate, pH 7.0), 0.1% SDS at 55°C. Blots were exposed on Kodak X- Ray films. For Western blot analysis, 20 µg of total cell lysate was analyzed by 12% SDS-PAGE, subsequently transferred to Hybond-C membrane. Specific proteins were detected using antibodies from Santacruz Biotechnology and electrochemiluminiscence (ECL) kit from GE Healthcare. c-Myc (9E-11) and Hog1p (yC-20) antibodies were used to detect myc-tagged GalBD-CaZF and *S. cerevisae* Hog1p respectively. Ponceau-S stained membranes were checked for equal protein loading. For semiquantitative RT-PCR, 1 µg RNA was converted to cDNA using Superscript Reverse Transcriptase (Invitogen). One-tenth of the cDNA product was used for PCR amplification. Amplified product was visible after 22 cycles. Primers used for CaZF are (5′ATGGCTTTAGAGTTAGAAGCTTTCAATTCTTC3′; 5′AGACGGATACAGTGTCGTTGAAGGCTGTGGATG 3′) and for actin are (5′ATGGATTCTGAGGTTGCTGCTTTGGTTATT3′; 5′AAAGAGTAACCACGTTCACTCAAGATCTTC3′).

### Glycerol estimation

Overnight grown yeast cells in YPGal medium were diluted to OD_600_ = 0.3 and grown for 4 h at 30°C. Then they were subjected to increased osmolarity (500 mM NaCl). For glycerol measurement, at time points indicated in the Figure. 7, 2 ml samples were taken, boiled for 15 min and then centrifuged to remove cellular debris. The supernatant was used for glycerol measurement by using Free Glycerol Reagent (Sigma, USA) according to the manufacturers' instructions. Assay was done with three independent experimental repeats. Glycerol accumulation was expressed in mg/ml (equivalent to triloen content).

### CTT1-*LacZ* β-galactosidase assay

Wild type BCY123 or *hog1* yeast cells were cotransformed with pYES-CTT1-*LacZ* construct containing a 800 bp (−137 to −937) upstream region of *CTT1* fused to *LacZ* and pGBKT7-*CaZF* construct expressing full length or truncated form of *CaZF* cDNA. Three independent transformants were grown to late log phase in SD medium without uracil and histidine. Cells were collected and re-suspended in YPD medium to an OD of 0.2–0.3. Growth was assumed until A_600_ of 0.5–0.7. Cells were harvested and resuspended in YPD with or without salt (500 mM NaCl) for 30 min. Cells from three independent transformants were collected and assayed for β-galactosidase activity as described above.

### Electrophoresis mobility shift assays

Recombinant CaZF protein was expressed in *E. coli* DH5α as GST-fused proteins and purified by GST-agarose columns. Gel-shift assays were performed with 10,000 c.p.m. of ^32^P-end-labeled probe A, a dimer of CTTTTTCAAGGGGATCACCGGTAAGGGGCCAAG (STRE sequences are underlined) or mutated probe M1, a dimer of CTTTTTCAAGG**a**GATCACCGGTAAGG**a**GCCAAG or probe M2, a dimer of CT**a**TTTCAAGGGGATCACCGGTAAGGGGCCAAG (mutated bases are in bold lower case letters). After incubation for 20 min at room temperature, the mixtures were subjected to electrophoresis in 8% polyacrylamide gel as described previously [70].

## Supporting Information

Figure S1 Detection of Hog1p. Hog1p was detected in S. cerevisiae BCY123 (WT) and the corresponding hog1 mutant strain by Western Blot with Hog1p-antibody (yC-20, sc-6815). Blotted membrane stained with Ponceau-S is shown for equivalent loading of protein (20 µg)
(9.28 MB TIF)

Table S1 Oligonucleotides used in this study
(0.07 MB DOC)
