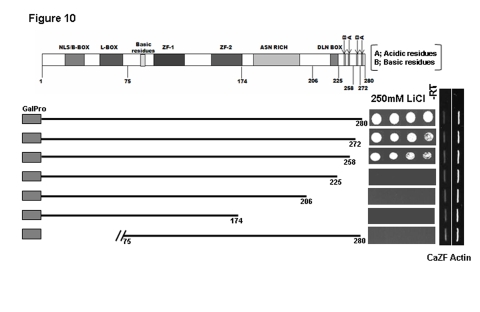# Correction: CaZF, a Plant Transcription Factor Functions through and Parallel to HOG and Calcineurin Pathways in *Saccharomyces cerevisiae* to Provide Osmotolerance

**DOI:** 10.1371/annotation/da3ad6f8-bc52-494d-9472-dd96c387c8fd

**Published:** 2009-06-19

**Authors:** Deepti Jain, Nilanjan Roy, Debasis Chattopadhyay

The published Figures 5, 6, and 10 contain errors in some panels. Please see the corrected figures here:

Figure 5, 

**Figure:**
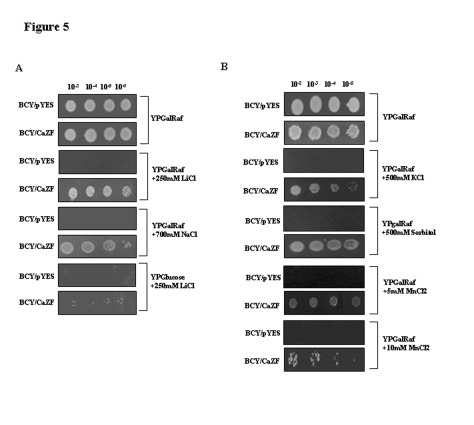


Figure 6, 

**Figure:**
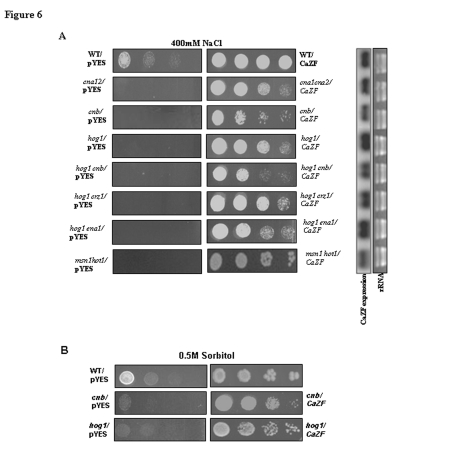


Figure 10, 

**Figure:**