# Supplementary figures and images for: CaZF, a Plant Transcription Factor Functions through and Parallel to HOG and Calcineurin Pathways in Saccharomyces cerevisiae to Provide Osmotolerance
Source: PLoS One. 2009 Apr 13;4(4):e5154. doi: 10.1371/journal.pone.0005154 (PMC2664467; doi:10.1371/journal.pone.0005154)

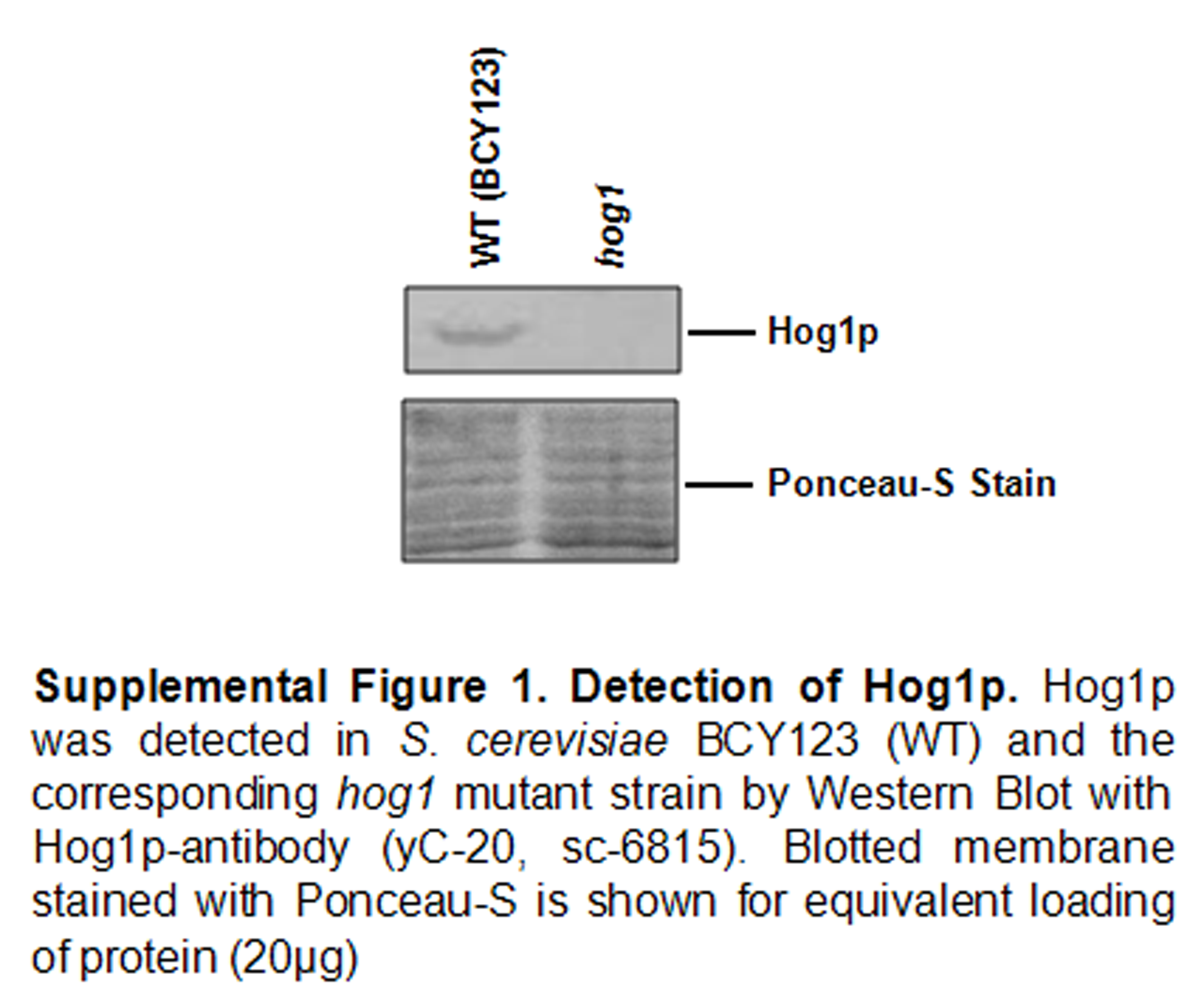

Supplement: Figure S1 — Detection of Hog1p. Hog1p was detected in S. cerevisiae BCY123 (WT) and the corresponding hog1 mutant strain by Western Blot with Hog1p-antibody (yC-20, sc-6815). Blotted membrane stained with Ponceau-S is shown for equivalent loading of protein (20 µg) (9.28 MB TIF) [file pone.0005154.s001.tif]
