# Supplementary material for: CaZF, a Plant Transcription Factor Functions through and Parallel to HOG and Calcineurin Pathways in Saccharomyces cerevisiae to Provide Osmotolerance
Source: PLoS One. 2009 Apr 13;4(4):e5154. doi: 10.1371/journal.pone.0005154 (PMC2664467; doi:10.1371/journal.pone.0005154)
Supplement: Table S1 — Oligonucleotides used in this study (0.07 MB DOC) [file pone.0005154.s002.doc]

**Supplemental Table S1: Oligonucleotides used in this study**

| **S. NO.** | **PRIMER** | **SEQUENCE (5’ TO 3’)** | **COMMENT(S)** |
| --- | --- | --- | --- |
| 1 | FCNA1 | CTGCCATAAAACACTCTCAACGCCAATGACAGAGCAGAAAGCCCTAAG | CNA1 knockout |
| 2 | RCNA1 | ATAAAAAAGAATAAAATGAGATTTACTACATAAGAACACCTTTGGTG | CNA1 knockout |
| 3 | FCNA2 | CCAGTACTTCTTCTTGAACCCGCAATGTCTGTTATTAATTTCACAGG | CNA2 knockout |
| 4 | RCNA2 | ATGTACAGTGGAAATAGGAGCTTTCTCTATTTCTTAGCATTTTTGACGA | CNA2 knockout |
| 5 | FCNB | CATAGAAGCATTTTTATTTCTTAAAATGTCTGTTATTAATTTCACAGG | CNB knockout |
| 6 | RCNB | TAAATGAATGAAGTGTCCCCTAGTCCTATTTCTTAGCATTTTTGACGA | CNB knockout |
| 7 | FHOG | AAAACTACAACTATCGTATATAATAATGTCTGTTATTAATTTCACAGG | HOG1 knockout |
| 8 | RHOG | GGTTAGGGACATTAAAAAAACACGTCTATTTCTTAGCATTTTTGACGA | HOG1 knockout |
| 9 | FMSN2 | TGCTCATAGAAGAACTAGATCTAAAATGTCTGTTATTAATTTCACAGG | MSN2 knockout |
| 10 | RMSN2 | TATCGAATTAAAAAAATGGGGTCTACTATTTCTTAGCATTTTTGACGA | MSN2 knockout |
| 11 | FMSN4 | TTTTCTTCTTATTAAAAACAATATAATGACAGAGCAGAAAGCCCTAGT | MSN4 knockout |
| 12 | RMSN4 | TTATTTGCTTTTGACCTTATTTTTTCTACATAAGAACACCTTTGGTGG | MSN4 knockout |
| 13 | FMSN1 | TGCTCATAGAAGAACTAGATCTAAAATGGCAAGTAACCAGCACATAGG | MSN1 knockout |
| 14 | RMSN1 | TATCGAATTAAAAAAATGGGGTCTATCACTTCAAAGTCTCTGGAATAT | MSN1 knockout |
| 15 | FHOT1 | TTTTCTTCTTATTAAAAACAATATAATGTCTGGAATGGGTATTGCGAT | HOT1 knockout |
| 16 | RHOT1 | TTATTTGCTTTTGACCTTATTTTTTCTATATTCCAGCAAGGCTCTCTT | HOT1 knockout |
| 17 | FCRZ1 | AGTTTCGTCAGACAGTACAAGGAAGATGACAGAGCAGAAAGCCCTAAG | CRZ1 knockout |
| 18 | RCRZ1 | TATTCAAAGCTTAAAAAAACAAAAACTACATAAGAACACCTTTGGTG | CRZ1 knockout |
| 19 | FENA1 | TCGTACACAGAATTGAAAATTTTCGATGACAGAGCAGAAAGCCCTAAG | ENA1 knockout |
| 20 | RENA1 | ATAGGGAGCACTTAATAGGCCCTGCCTACATAAGAACACCTTTGGTG | ENA1 knockout |
| 21 | RHIS3 | AGTTCGACAACTGCGTACGG | Diagnostic PCR |
| 22 | RTRP1 | AGT AGTATGTTGCAGTCTTTTG | Diagnostic PCR |
| 23 | FCTT1 | TACTCTCTACAAAACGGTTTTCCGTAC | CTT1 probe |
| 24 | RCTT1 | ATAATCCCAGTATATGGTAGTGTCCTG | CTT1 probe |
| 25 | FHSP12 | ATGTCTGACGCAGGTAGAAAAGGATTC | HSP12 probe |
| 26 | RHSP12 | ACTTCTTGGTTGGGTCTTCTTCACCGT | HSP12 probe |
| 27 | FGPD1 | ATGTCTGCTGCTGCTGATAGATTAAAC | GPD1 probe |
| 28 | RGPD1 | AAGAGGATAGCAATTGGACACCTTTAG | GPD1 probe |
| 29 | FGPP2 | GGATTGACTACTAAACCTCTATCTTTG | GPP2 probe |
| 30 | RGPP2 | GCCATTCCTGCCCTTCAGATATGGTTC | GPP2 probe |
| 31 | FENA1 | ACAAGTCTAACTGAAGGTTTGACCCAAG | ENA1 probe |
| 32 | RENA1 | TAACGGCACCTAAAGCTTCCAGAGAATC | ENA1 probe |
| 33 | FSTL1 | ATAAGCAGAACCAGTCACTGGGGACTTACG | STL1 probe |
| 34 | RSTL1 | AGTAGATAAGGCGTAGATTGTTGCGAAGAC | STL1 probe |
| 35 | FPYFL | CCCTCGAGATGGCTTTAGAGTTAGAAGCT | CaZF Cloning |
| 36 | RPYFL | GCTCTAGACTACACCGTTTCATCATCATG | CaZF Cloning |
| 37 | F(-Asn) | CCCTCGAGATGGCTTTAGAGTTAGAAGCT | CaZF Cloning |
| 38 | R(-Asn) | GCTCTAGACTATTCGTAGTGGCATCGTTTGT | CaZF Cloning |
| 39 | F(-DLNL) | CCCTCGAGATGGCTTTAGAGTTAGAAGCT | CaZF Cloning |
| 40 | R(-DLNL) | CGGGATCCGCCCTCAGATATTGTGATTCCACTGCT | CaZF Cloning |
| 41 | F(-Basic Region) | CCCTCGAGATGGCTTTAGAGTTAGAAGCT | CaZF Cloning |
| 42 | R(-Basic Region) | CGGGATCCAGGCGCAGGTAGATTGAGGTCAAATCC | CaZF Cloning |
| 43 | F(-KRPR) | CCCTCGAGATGGCTTTAGAGTTAGAAGCT | CaZF Cloning |
| 44 | R(-KRPR) | CGGGATCCGCTTTCCACCTCCTGTTCACCGATTCC | CaZF Cloning |
| 45 | F(-DDE) | CCCTCGAGATGGCTTTAGAGTTAGAAGCT | CaZF Cloning |
| 46 | R(-DDE) | CGGGATCCCCAAAAACACACGTGGCCTTTTGGCGGT | CaZF Cloning |
| 47 | F(-TD) | (NdeI/XhoI)ATGGTGAAGCTGAATCACCGCTGC | CaZF Cloning |
| 48 | R(1/179) | CGGGATCCGATACCTCCTTCGTAGTGGCA | CaZF Cloning |
| 49 | F(198/280) | CGGGATCCAGCAGTGGAATCACAATATCTGAG | CaZF Cloning |
| 50 | FGFP | CATGCCATGGCTTTAGAGTTAGAAGCT | CaZF Cloning |
| 51 | RGFP | GAAGATCTTGCACCGTTTCATCATC | CaZF Cloning |
| 52 | FActin | CCACGAGACAACATTTAACTC | DNA-array |
| 53 | RActin | TATTCTGCCTTTGCAATCCAC | DNA-array |
| 54 | FNPTII | TTTTCTCCCAATCAGGCTTG | DNA-array |
| 55 | RNPTII | TCAGGCTCTTTCACTCCATC | DNA-array |
